# Label‐Free Sorting of Human Mesenchymal Stem Cells Using Insulating Dielectrophoresis

**DOI:** 10.1002/elps.70001

**Published:** 2025-07-24

**Authors:** Zuri A. Rashad, Kiara L. Lacy, Emmanuel Egun, Jazmine S. Moore, Tayloria N. G. Adams

**Affiliations:** ^1^ Department of Chemical and Biomolecular Engineering Samueli School of Engineering University of California Irvine California USA; ^2^ Sue & Bill Gross Stem Cell Research Center University of California Irvine California USA; ^3^ Department of Anatomy and Neurobiology School of Medicine University of California Irvine California USA; ^4^ Department of Biomedical Engineering Samueli School of Engineering University of California Irvine California USA; ^5^ Department of Materials Science and Engineering Samueli School of Engineering University of California Irvine California USA

## Abstract

Human mesenchymal stem cells (hMSCs) are a multipotent yet heterogeneous cell population with immunosuppressive and regenerative properties, making them highly promising for stem cell therapies targeting metabolic diseases. However, the inherent heterogeneity of hMSCs presents challenges for producing consistent therapeutic outcomes, emphasizing the need to isolate functionally distinct subpopulations. In this study, we employed insulating dielectrophoresis (DEP) via a trap‐and‐release sorting strategy to generate and characterize subpopulations of adipose tissue (AT)‐derived hMSCs. Voltage and frequency parameters were systematically tuned, revealing that higher voltages increased the percentage of trapped cells, while higher frequencies had less impact. Sorted cells underwent a 14‐day adipogenic differentiation process, assessed by Oil Red O staining. Our results demonstrated that untrapped cell populations generated at lower voltage and frequency thresholds exhibited enhanced adipogenic differentiation compared to unsorted controls. These findings suggest that DEP can be leveraged to isolate progenitor cells within hMSC populations, enabling the production of homogeneous cell subsets with targeted functional potential. This work highlights the utility of insulating DEP for addressing hMSC heterogeneity and advancing the development of stem cell therapies.

## 1 Introduction

Human mesenchymal stem cells (hMSCs) are vital to cell therapy research because of their ability to act as a self‐repair system in the body [1]. hMSCs can differentiate into multiple cell types, interact with immune cells, and secrete cell signaling molecules that modulate the cell microenvironment [1]. Although hMSCs have demonstrated safety and utility in many early‐stage clinical trials [2], their therapeutic outcomes remain inconsistent, with many therapies failing to progress beyond late‐stage trials or even beyond the preclinical phase [3]. This variability arises from the inherent heterogeneity of hMSCs [4], as cultures expanded for transplantation often contain stem cells, partially differentiated progenitor cells, and fully differentiated cells.

Adipose tissue (AT)‐derived hMSC transplantations have shown promise in improving adipocyte function and recovery in animal models of a variety of metabolic diseases, including insulin resistance [5], diabetes [6], and hyperlipidemia [7]. For instance, Lee et al. injected AT‐hMSCs into the AT of obese rats, and the presence of the cells suppressed the formation of obesity‐related metabolic syndromes such as glucose intolerance [7]. Moreover, Kono et al. used AT‐hMSCs containing a low dose of streptozotocin to proliferate β cells within the bodies of immunocompromised mice and reduced the likelihood of developing type 1 diabetes [6]. In these studies, the AT‐hMSCs were transplanted as heterogeneous cell populations, which remains standard practice in hMSC transplantation research. Heterogeneity in hMSC cultures can be addressed using fluorescent activated cell sorting (FACS), a label‐based cell sorting method. Yet, this approach impairs the biological activity of cells [8], exposes them to high shear stress that impacts viability [9], and requires long processing times, typically around 4 h [9]. Therefore, there is a compelling need for a cell sorting strategy that preserves cell function and viability while leveraging the cells’ intrinsic properties to separate cells into distinct subpopulations.

One emerging label‐free cell sorting technique is dielectrophoresis (DEP), which utilizes the inherent electrical properties of cells to produce subpopulations without modification. With DEP, we found that the electrical signatures of homogeneous cells and heterogeneous hMSCs are unique [10]. We also found that the electrical signatures of AT‐hMSCs and bone marrow–derived hMSCs are different and highlight differences in the differentiation potential of hMSCs, with cytoplasm conductivity serving as a potential biomarker of hMSC fate [10]. More recently, we demonstrated that DEP‐based electrical phenotyping detects shifts in membrane capacitance and cytoplasm conductivity with hMSC aging and that these properties correlate with osteogenic and adipogenic gene expression [11]. Others have found that membrane capacitance indicates the cell fate of neural stem and progenitor cells [12], human adipose–derived stem cells [13], and human embryonic stem cells differentiated toward a mesenchymal stem cell–like phenotype [14]. Furthermore, DEP has been used to enrich osteoblasts or hMSCs from heterogeneous mixtures [15, 16]. These studies collectively demonstrate DEP's potential for isolating homogeneous subpopulations of stem cells at the progenitor level based on their distinct electrical properties.

Generally, DEP uses the polarizable characteristics of particles to move them within nonuniform electric fields due to the differences in particle and medium polarizability. Although cells are negatively charged, DEP does not require particles to carry a net charge, unlike traditional electrophoresis. This creates more versatility for manipulation of various particles, including cells [17], nanoparticles [18], and biomolecules [19]. With DEP, a force is enacted on cells, which is given by FDEP=2πr3εmRe[K(ω)]∇∣E∣2, where $F_{\mathrm{DEP}}$ is the dielectrophoretic force, *r* is the radius of the cell as a spherical particle, $\varepsilon_{m}$ is the permittivity of the surrounding medium, Re[K(ω)] is the real part of the Clausius–Mossotti factor, which depends on the frequency of the electric field ω, and $\nabla |E{|}^{2}$ is the gradient of the squared magnitude of the electric field *E* [20]. Depending on the sign of Re[K(ω)], particles may experience positive DEP (attraction to regions of higher field strength) or negative DEP (repulsion from these regions).

DEP can be implemented in various modes for cell sorting, with two prominent approaches: traditional DEP and insulating DEP. Unlike traditional DEP, which relies on metal electrodes to generate nonuniform electric fields, insulating DEP utilizes insulating structures to achieve the same effect. Specifically, the electric field gradients are generated by embedding barriers in the microchannel geometry. The same DEP force principles apply, but the field's spatial non‐uniformity is achieved through the design of the posts. Insulating DEP devices, including constriction channels, pillar arrays, and obstacle‐based designs, have been extensively reviewed by Lapizco‐Encinas [21], highlighting their impact on generating nonuniform electric fields. These designs are tailored for applications like particle separation, trapping, or concentration and are often optimized for parameters such as throughput, specificity, and ease of fabrication. A notable example of commercialized insulating DEP technology is the CytoChip^TM^, a platform developed by CytoRecovery Inc., which utilizes arrays of insulating pillars to enable label‐free cell sorting, addressing research needs such as the enrichment of stem cell subpopulations. One advantage of the CytoChip is it avoids direct electrode contact with cell suspension, reducing issues like electrolysis or electrode fouling. This also helps maintain cell viability. Insulating DEP has been widely used for cancer cell isolation, including enriching breast cancer cells [22], leukemia cells [23], and circulating tumor cells [24]. Insulating DEP is a beneficial mode of DEP to implement for the isolation of therapeutically relevant subpopulations of hMSCs.

In this study, we used insulating DEP via the CytoChip to generate subpopulations of AT‐hMSCs. Trapping behavior and viability were systematically characterized through voltage and frequency sweeps prior to full sorting experiments. Our findings revealed that specific sorting conditions (i.e., combinations of voltage and frequency) consistently yielded 30% of cells trapped and 70% of cells untrapped. Interestingly, higher voltages yielded the highest viability among the trapped cells, but the overall health of the trapped cells was not suitable for downstream analyses. Additionally, differences were observed in velocity for the trapped and untrapped cells. Focusing on the untrapped cell population, we found differences in the adipogenic potential compared to the unsorted controls, confirming successful isolation of functionally distinct subpopulations of AT‐hMSCs.

## 2 Materials and Methods

### 2.1 Culture of hMSCs and Preparation for DEP Sorting

AT‐hMSCs obtained from ATCC (PCS‐500‐011) were grown and passaged in proliferation media as previously described [10]. All AT‐hMSCs were maintained in an incubator at 37°C and 5% CO_2_. Once the AT‐hMSCs reached 80% confluency, they were prepared for DEP sorting. All experiments were completed on AT‐hMSCs at Passages 5, 6 and 7.

In preparation for DEP sorting, 80% confluent AT‐hMSCs were trypsinized for approximately 5 min and neutralized with proliferation media. The neutralized cells were collected in a 15 mL conical tube and centrifuged at 290 × *g* for 5 min. Next, the cells were resuspended in low conductivity DEP buffer (0.725% (v/v) RPMI, 8.5% (w/v) sucrose, and 0.3% (w/v) glucose) at ∼100 µS/cm. The cells were washed with the DEP buffer three times, counted, and resuspended at a final concentration of 1 × 10^6^ cells/mL. Cell radius and circularity were assessed from hemocytometer images of cells in suspension using ImageJ.

### 2.2 Preparation of the CytoChip for Cell Sorting

The insulating DEP device, the CytoChip (Type A chip, CytoRecovery, Blacksburg, VA, USA), was used for cell sorting. The CytoChip features a microchannel consisting of two inlets that converge to form a Y‐shaped junction. These inlets connect to a main microchannel with two cell filter regions and a DEP‐trapping area, oriented in a hexagonal cross‐section arrangement. The DEP trapping region is 3 mm in width, 12.24 mm in length, and 50 µm in height with a large array of micropillars that create electric field gradients using external electrodes. The external electrodes are metal electrodes with a height of 50 µm, which are on the outside edges of the DEP trapping region. A thin membrane, 14 µm thick, prevents cell contact with the external electrodes. Downstream of the main microchannel, the flow splits into two outlets. The device is constructed from two PDMS slabs forming the microchannel, with a cell filter region to control cellular flow into and out of the device and the DEP trapping zone. A schematic of the device is available in previously published work [25, 26]. Prior to cell separation, all CytoChip devices were screened for defects that could influence DEP trapping. The CytoChip was then prepared for cell sorting following a series of steps.

First, the CytoChip was primed with 70% ethanol via one outlet hole. The device was checked to ensure no bubbles were present. Next, a syringe pump containing a 1 mL syringe of low conductivity DEP buffer with 0.25% Pluronic F‐127 was inserted into one of the inlets. The syringe pump flow rate was set to 10 µL/min for 2.5 min, and the ethanol was removed from the device. During ethanol removal, all liquid that accumulated at the outlet and second inlet was carefully collected. After 2.5 min, the DEP buffer with 0.25% Pluronic F‐127 was set to 0.3 µL/min. A syringe containing the cell suspension was then connected to the second inlet, with the flow rate set to 5 µL/min for 20 s before being reduced to 1.2 µL/min. After 1 min passed, the fluid flow was checked on the microscope to confirm stability, ensuring no pulsed or stopped fluid flow.

### 2.3 hMSC Sorting With Insulating DEP via the CytoChip

A trap‐and‐release cell sorting strategy was implemented using the CytoChip. A specific voltage and frequency were selected to trap a percentage of cells along the insulating posts, whereas untrapped cells flowed past the posts. The general trapping procedure involved the following steps: setting the voltage and frequency, turning on the electric field, and trapping cells along the insulating posts for 20 min, whereas untrapped cells were collected at the outlet in 10 µL increments in a 1.5 mL microcentrifuge tube. After 20 min of untrapped cell collection, the flow rate of the cell suspension was reduced to 0.3 µL/min, and the flow rate of the DEP buffer was increased to 1.2 µL/min, allowing all untrapped cells to be collected; the collection of untrapped cells lasted 3–5 min. Next, the electric field was turned off, and the DEP buffer flow rate was set to 5 µL/min for 3 min to release and collect the trapped cells in a 1.5 mL microcentrifuge tube. Once the trapped cells were collected, the flow rates of the cell suspension and DEP buffer were returned to 0.3 and 1.2 µL/min, respectively, for additional cell collection. To optimize the frequency and voltage parameters for cell sorting, a frequency sweep (100, 200, 300, 400, and 500 kHz) and voltage sweep (100, 200, 300, 400, 500, and 550 V_pp_) were conducted. For the full cell sorting experiment, the trap‐and‐release process was repeated three times, with the collected cells pooled together, checked for viability with trypan blue, and subsequently plated for proliferation and differentiation studies. Additional viability assessments were performed on control, trapped, and untrapped cells sorted at 200 kHz and 200 V_pp_, including post‐sort passaging. These experiments used bone marrow–derived hMSCs. Unsorted control cells were collected in a 1.5 mL microcentrifuge tube and similarly used for proliferation and differentiation studies.

### 2.4 hMSC Differentiation

**FIGURE 1:**
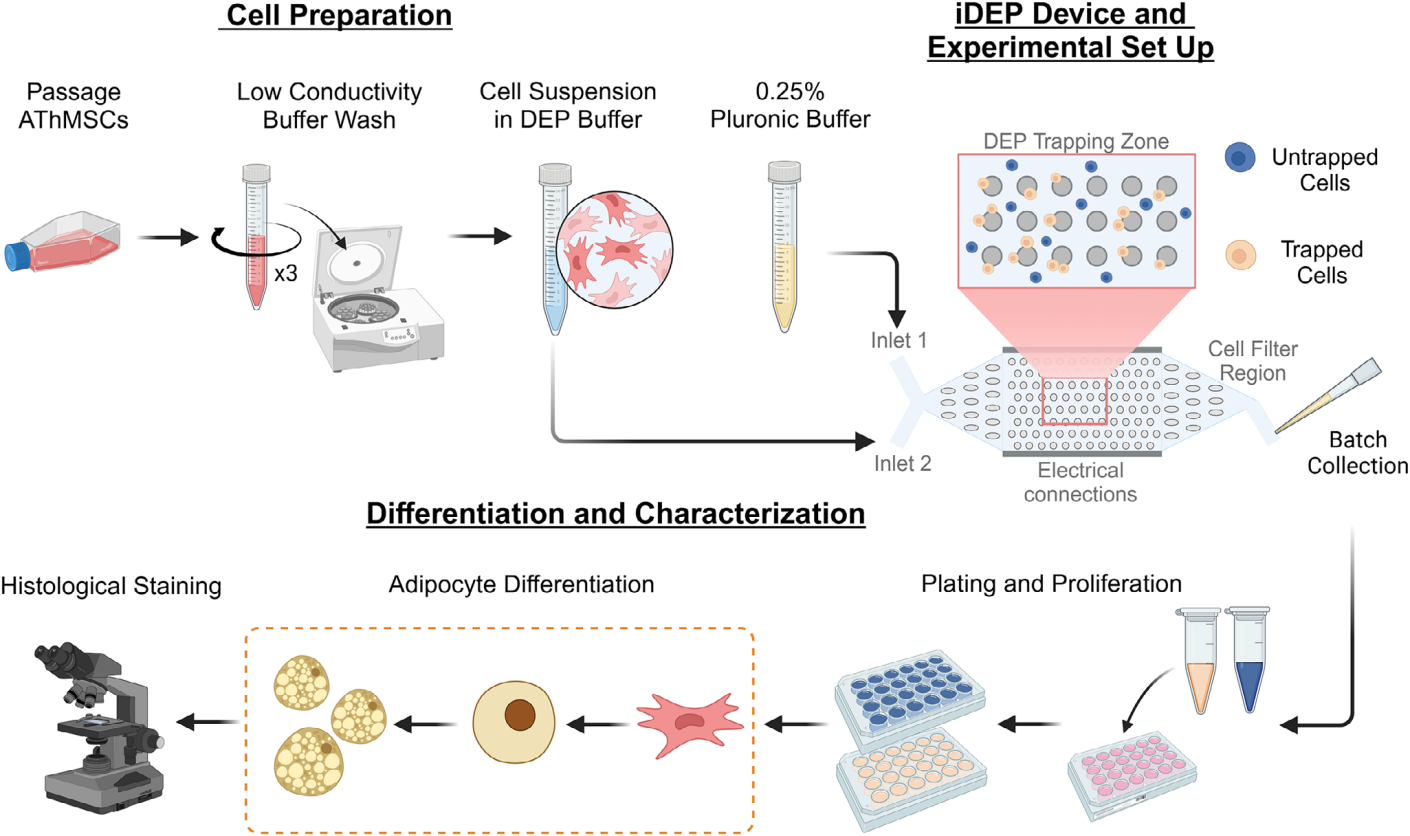
General workflow for preparing AT‐hMSCs for insulating DEP cell sorting. Cells were suspended in a low conductivity DEP buffer (∼100 µS/cm) at a concentration of 1 × 10^6^ cells/mL. The insulating DEP device (CytoChip) contained two inlets leading to a cell filter region, followed by a DEP trapping zone and a second filter region before the outlet. DEP cell sorting was performed using a trap‐and‐release method: the untrapped population was collected first, followed by the trapped population after the electric field was turned off. Cells were plated, expanded, and set up for a 14‐day adipocyte differentiation. Oil Red O histological staining was used to evaluate adipocyte differentiation. AT, adipose tissue; DEP, dielectrophoresis; hMSCs, human mesenchymal stem cells.

The overall experimental workflow, including hMSC sorting, viability assessment, and downstream differentiation, is summarized in Figure 1. After sorting, the collected trapped, untrapped, and control cells were diluted up to 250 µL using MSC proliferation media and counted. The cells were plated at similar densities in a 48‐well plate and returned to the incubator for cell proliferation. Forty‐eight hours after plating, the MSC proliferation media was replaced with fresh proliferation media. Subsequent proliferation media replacements were performed every 48 h until the cells reached 80% confluency.

Once the cells reached 80% confluency, they were either passaged once before differentiation or directly differentiated. Differentiation was performed either in wells coated with 0.02% gelatin. Forty‐eight hours after plating, the MSC proliferation media was replaced with adipocyte differentiation media as previously described [10]. After differentiation, the cells were fixed with 4% paraformaldehyde for 15 min at room temperature, rinsed three times with 1× DPBS, and stored in 0.05% sodium azide in 1× DPBS at 4°C.

Adipocyte differentiation was assessed using Oil Red O staining to visualize lipid accumulation. The Oil Red O stock solution was prepared by dissolving 0.5% (w/v) Oil Red O in pure isopropanol and mixing it with Milli‐Q water at a 3:2 ratio. The solution was sterile filtered. The control, trapped, and untrapped cells were incubated in Oil Red O for 10 min at room temperature. After staining, the Oil Red O solution was aspirated, and the cells were rinsed three times with Milli‐Q water. All cell samples were imaged using a digital Keyence microscope.

### 2.5 Statistical Analysis

The voltage sweep, frequency sweep, and viability tests were performed three times (biological repeats *n* = 3). Statistical analysis was completed using a two‐sample *t*‐test with variance. Statistical significance is denoted by *p < 0.1, **p < 0.01, ***p < 0.001, ****p < 0.0001, and not significant (n.s.).

## 3 Results

**FIGURE 2:**
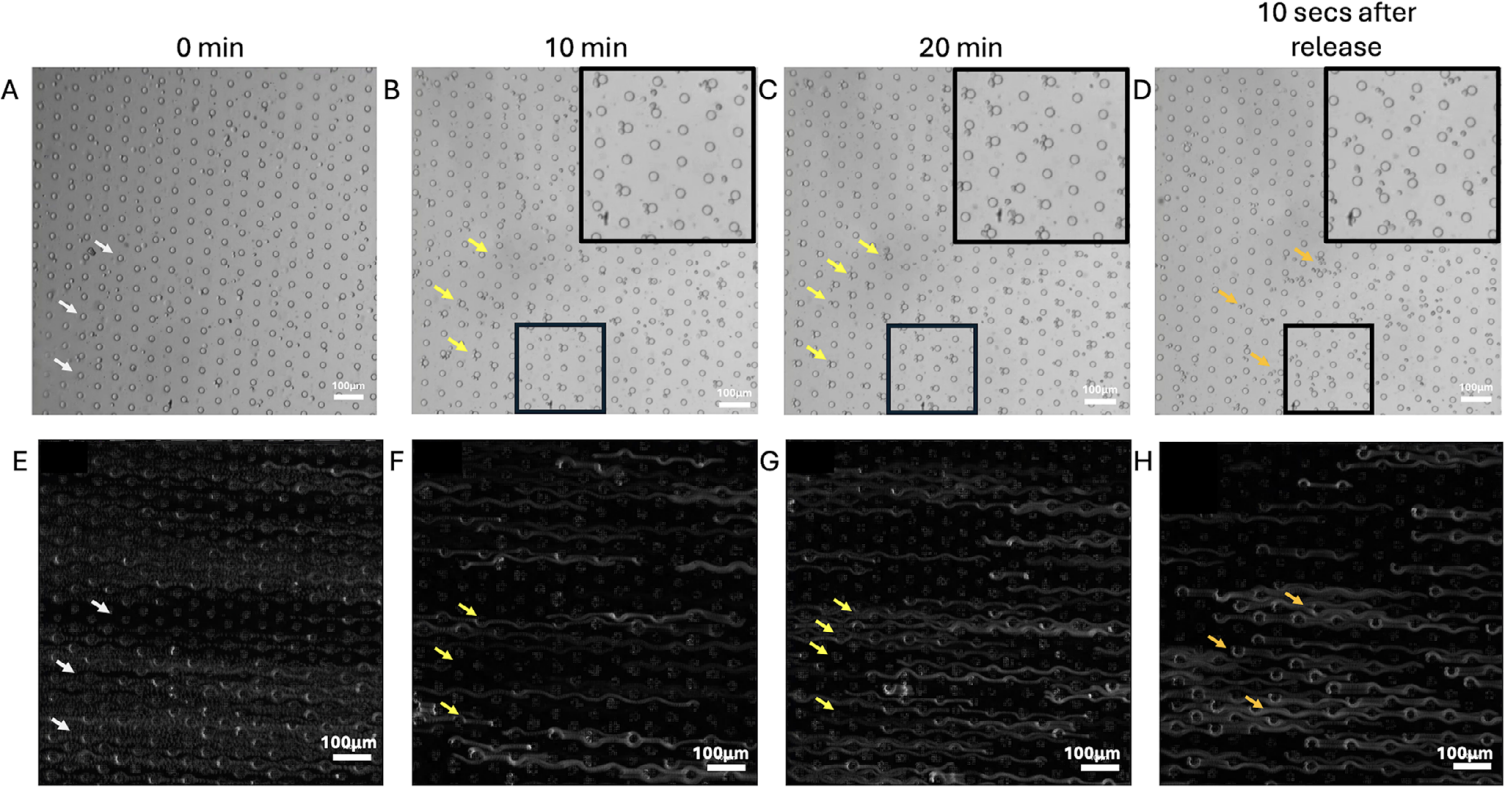
Time‐lapsed images of AT‐hMSCs undergoing cell sorting at 400 kHz and 500 V_pp_. (A) Cells at *t* = 0 min (electric field applied), (B) *t* = 10 min, (C) *t* = 20 min, and (D) 10 s after the electric field was turned off (trapped cells released). The insets provide views of cells along the insulating posts. (E–H) Standard deviation Z‐projections at corresponding time points, generated from 30‐s image slices, highlight cell movement and trapping patterns.

In this work, insulating DEP via the CytoChip was utilized to detect and separate subpopulations within AT‐hMSCs. Using the workflow depicted in Figure 1, we sorted AT‐hMSCs into trapped and untrapped subpopulations using the CytoChip. Prior to insulating DEP sorting, we measured the size and circularity of suspended AT‐hMSCs, finding an average cell radius of 10.39 ± 2.62 µm and an average circularity of 0.48 (Figure S1). Figure 2A–C presents time‐lapse images from a representative cell sorting experiment at 400 kHz and 500 V_pp_, demonstrating the behavior of trapped and untrapped cells within the device over 20 min, the duration of one batch collection. As the time of the electric field application increased, the number of cells trapped along the insulating posts increased steadily up to ∼15 min. After this, the electric field was maintained for an additional 5 min to maximize the collection of untrapped cells at the outlet. Following the completion of untrapped cell collection, the electric field was turned off, releasing the trapped cells from the insulating posts, as shown in Figure 2D. White, yellow, and orange arrows in Figure 2A–D indicate empty insulating posts, trapped cells, and the release of trapped cells, respectively. Video S1 illustrates the movement of two representative cells during the sorting process: one cell that becomes trapped (blue) and one that remains untrapped (green). The velocities of the trapped and untrapped cells were measured as 3.8 and 7.8 µm/s, respectively, highlighting the distinct behaviors of these subpopulations under the applied electric field.

To provide another perspective of cell sorting within the CytoChip, we applied a standard deviation Z‐projection analysis in ImageJ to the time‐lapse images, as shown in Figure 2E–H. The standard deviation Z‐projection analysis highlights intensity variations in the images, which reflect cell movement and positioning over time. In Figure 2E–G, the higher intensity along the insulating posts corresponds to trapped cells, whereas the streaks of lower intensity correspond to untrapped cells flowing past the insulating posts. In Figure 2H, the release of trapped cells from the insulating posts is visualized by intensity around the curvature of the posts and along the flow pathway previously occupied by the untrapped cells.

**FIGURE 3:**
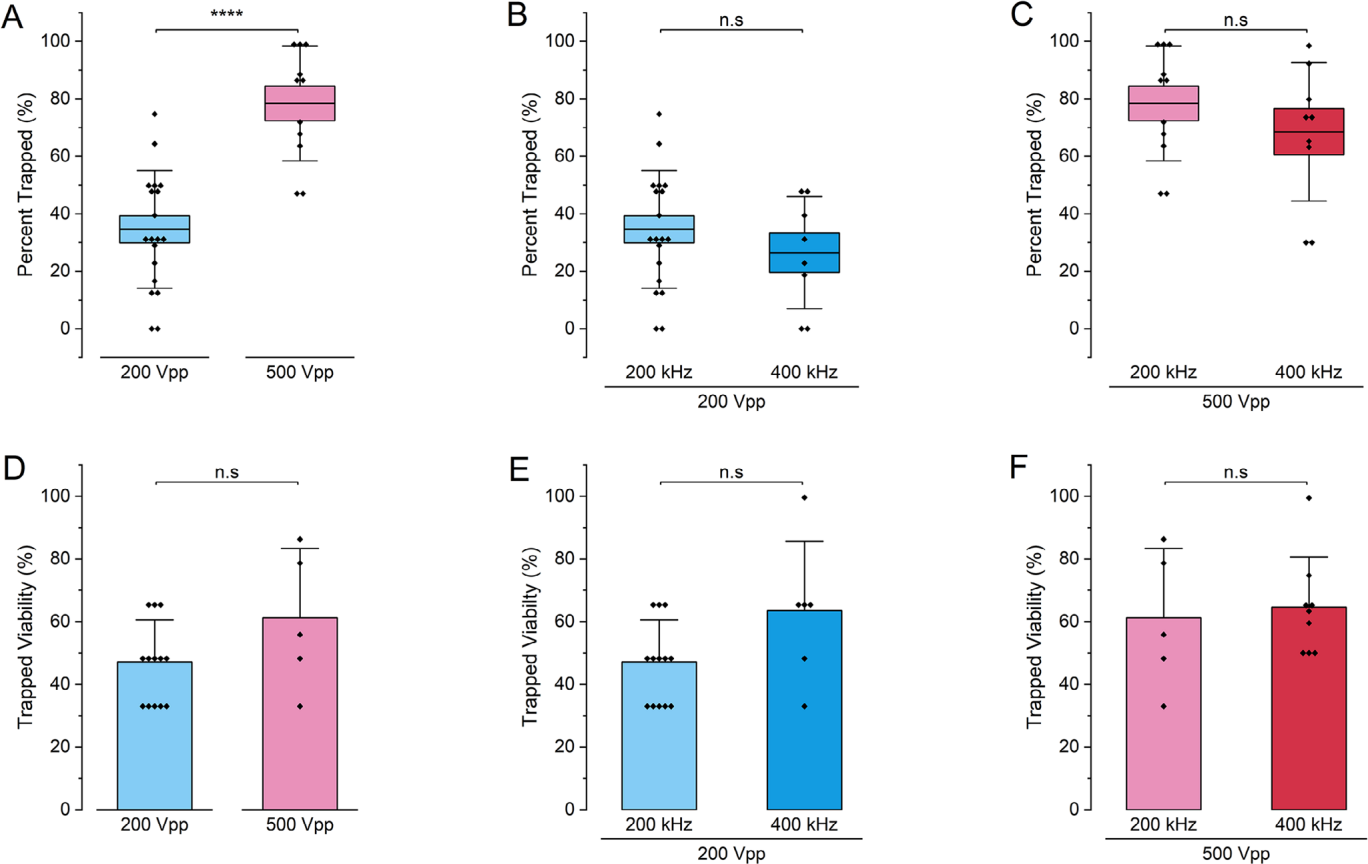
Trapping behavior and viability of AT‐hMSCs with insulating DEP. Percentage of trapped cells at varying (A) voltages and (B and C) frequencies. (D–F) Cell viability for each voltage and frequency condition. Statistical significance was determined using a two‐sample *t*‐test with variance (****p < 0.0001; n.s., not significant).

To better understand the heterogeneity and DEP behavior of our AT‐hMSCs, we conducted voltage and frequency sweeps to observe the percentage of cells trapped with each condition (Figure 3). Unlike standard insulating DEP sorting, which typically employs high voltages to generate localized nonuniform electric fields for trap‐and‐release strategies [27], we explored lower (i.e., 200 V_pp_) and higher (500 V_pp_) voltages. Two frequencies, 200 and 400 kHz, were chosen to observe the impact on cell behavior, as there was a significant number of cells showing both positive and negative DEP in the device. The voltage sweep revealed a significant difference in the percentage of trapped cells along the insulating posts at 200 V_pp_ and 500 V_pp_ at 200 kHz (Figure 3A). Specifically, ∼78.5% of cells were trapped at 500 V_pp_, whereas ∼32.7% of cells were trapped at 200 V_pp_, with statistical significance (****p < 0.0001). In contrast, the frequency sweep showed similar percentages of trapped cells at 200 and 400 kHz, with approximately 26.5% trapping at 200 V_pp_ and 68.6% trapping at 500 V_pp_ (Figure 3B,C), with no statistically significant differences. Cell viability was also measured for each condition (Figure 3D–F). Interestingly, trapped cells at 200 V_pp_ and 200 kHz had the lowest viability (45.6%), whereas trapped cells under other conditions—200 V_pp_ and 400 kHz (63.5%), 500 V_pp_ and 200 kHz (61.2%), and 500 V_pp_ and 400 kHz (64.7%)—showed higher viabilities. However, these trends were not statistically significant. Conversely, the viability of untrapped cells showed the opposite trend, with 200 V_pp_ and 200 kHz yielding the highest viability compared to the other conditions (Figure S2).

To further evaluate viability under these sorting conditions, we conducted supplemental experiments at 200 kHz and 200 V_pp_ using bone marrow–derived hMSCs. Viability was 85% for both trapped and untrapped cells, compared to ∼91% for unsorted controls. After post‐sort passage one (PSP1), viability remained high, with ∼83% for trapped cells and ∼87.5% for untrapped cells (Figure S3).

**FIGURE 4:**
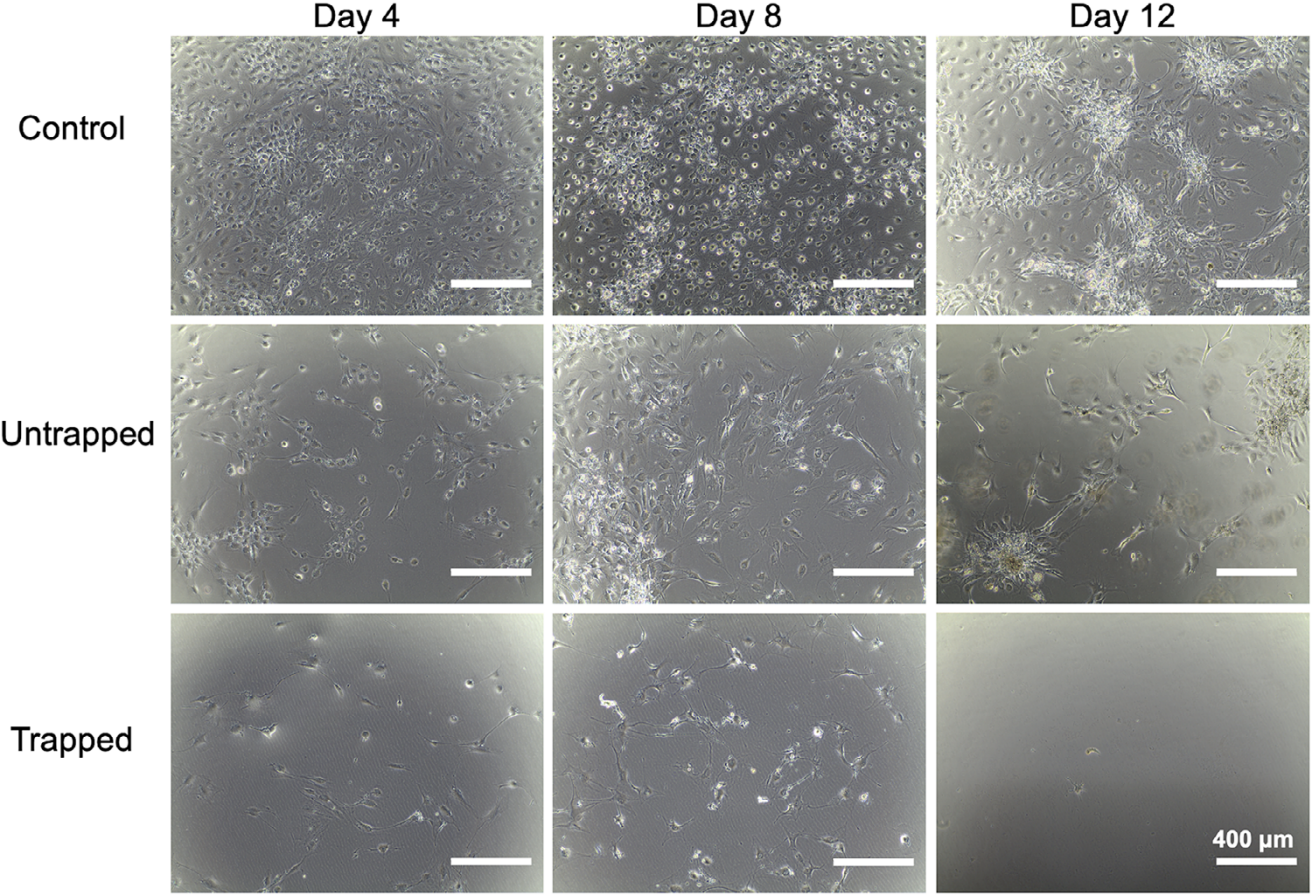
Representative images of 14‐day adipogenic differentiation of control (top), untrapped (middle), and trapped (bottom) cells sorted using insulating DEP.

The trapped and untrapped subpopulations of AT‐hMSCs, along with control cells, underwent adipocyte differentiation for 14 days (Figure 4). During adipogenesis, cells form white fat deposits and cluster together over time, eventually creating pockets of AT. In the control and untrapped populations, adipogenic changes became apparent around Day 8 of differentiation, with large fat deposits forming by Day 12 and surrounding cells clustering around these adipocyte formations. In contrast, when the trapped cells were plated, few adhered despite being seeded at a density similar to the control and untrapped populations. Consequently, trapped cells did not form white fat deposits. However, their morphology transitioned from round to spindle‐like around Day 4, resembling the morphology of control and untrapped cells that did not differentiate into adipocytes.

**FIGURE 5:**
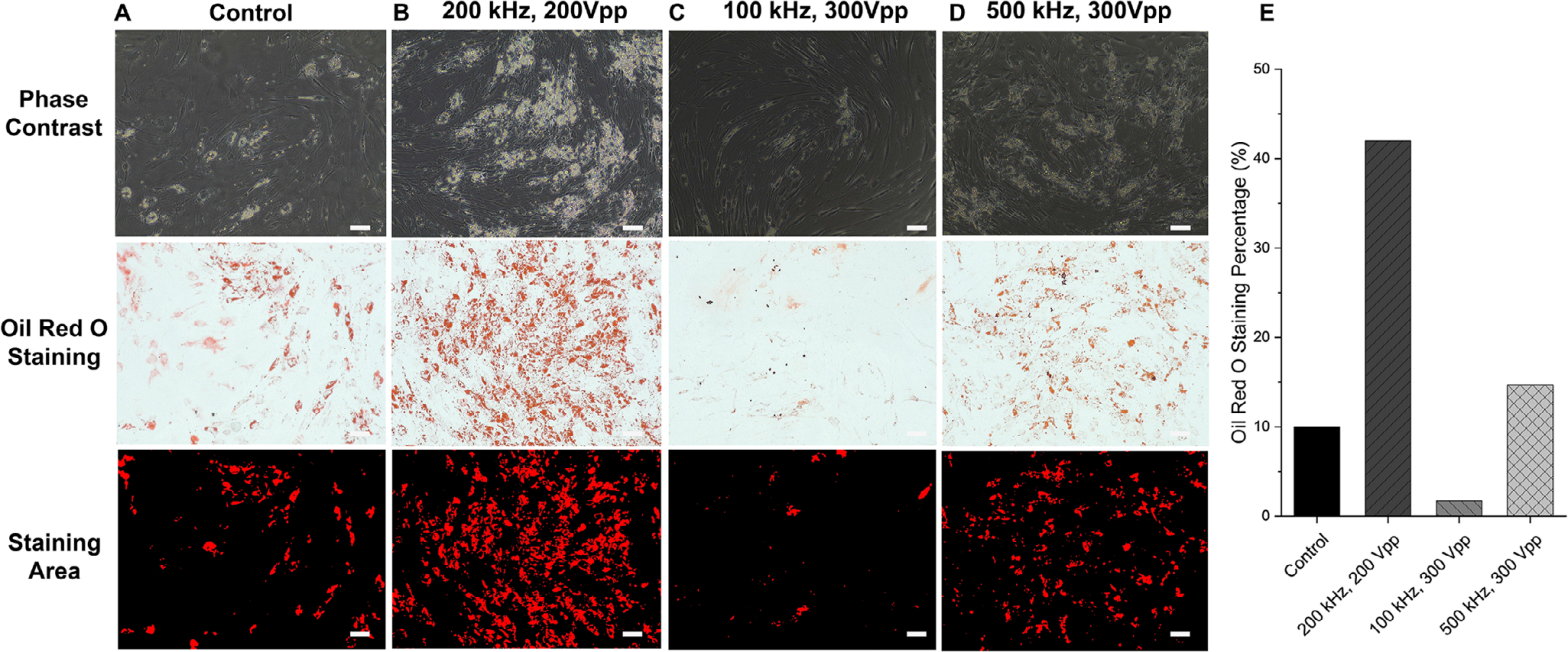
Assessment of the adipogenic potential in (A) control cells, (B) untrapped cells sorted at 200 kHz and 200 V_pp_, (C) untrapped cells sorted at 100 kHz and 300 V_pp_, (D) untrapped cells sorted at 500 kHz and 300 V_pp_, and (E) quantification of staining area. The top row displays phase contrast images, the middle displays Oil Red O staining of cells differentiated for 14 days, and the bottom displays images after processing for area coverage. Scale bar = 100 µm.

On the basis of our findings in Figures 3 and 4, we were interested in characterizing the adipogenic potential of untrapped cells at voltage and frequency combinations yielding ∼30% trapped and ∼70% untrapped cells and comparing them against unsorted control cells (Figure 5). More specifically, we sorted untrapped subpopulations of AT‐hMSCs at 200 kHz and 200 V_pp_, 100 kHz and 300 V_pp_, and 500 kHz and 300 V_pp_, assessing their adipogenic potential with phase contrast images and Oil Red O staining. All untrapped cell samples differentiated into adipocytes, visualized by positive Oil Red O staining. Among these, untrapped cells sorted at 200 kHz and 200 V_pp_ along with 500 kHz and 300 V_pp_ exhibited greater differences in adipogenic potential compared to the control. Figure 5 also presents quantification of Oil Red O staining, showing increased area coverage for untrapped cells sorted at 200 kHz and 200 V_pp_ and at 500 kHz and 300 V_pp_, compared to control cells; this increase was not observed for the 100 kHz and 300 V_pp_ condition.

## 4 Discussion

AT‐hMSCs have shown particular promise in addressing metabolic diseases through their ability to modulate the microenvironment and restore adipocyte function. Despite their potential, the therapeutic efficacy of hMSCs is often limited by their inherent heterogeneity, highlighting the importance of developing cell sorting methods that preserve cell viability and functionality while isolating therapeutically relevant subpopulations.

In this study, we used insulating DEP via the CytoChip as a label‐free method to sort subpopulations of hMSCs and address heterogeneity. The trap‐and‐release cell sorting strategy allowed for effective isolation of trapped and untrapped cells under various voltage and frequency conditions. This approach is supported by literature demonstrating that the CytoChip geometry generates strong electric field gradients at the edges of the insulating posts, with ∇|*E*|^2^/|*E*| values reaching ∼10^10^ V/m—sufficient to induce effective DEP trapping forces [28]. One advantage of this system is its moderate throughput (∼50,000 cells/min), which compares favorably to traditional DEP devices and demonstrates potential for scalable applications in stem cell research.

To characterize DEP behavior during sorting, we assessed the velocity of cells within the microchannel. Trapped cells moved more slowly than untrapped cells, as DEP forces exerted a stronger influence on their motion than the volumetric flow of the buffer solution. Standard deviation Z‐projections provided a dynamic view of cell behavior, clearly distinguishing trapped and untrapped populations. We further evaluated sorting efficiency across different voltage and frequency conditions. The CytoChip device enabled targeting specific subpopulations using voltage and frequency combinations, yielding trapping efficiencies of ∼30% for trapped cells and ∼70% for untrapped cells. With the voltage sweep, we observed a significant difference between the percentage of trapped and untrapped cells, corresponding with DEP theory, as the DEP force shows that increasing the electric field strength directly increases the strength of the DEP, which leads to a stronger positive DEP response.

Interestingly, we observed lower viability of trapped cells at lower voltages compared to higher voltages, which was unexpected, as prior studies have reported increased cell death at higher voltages and frequencies [28, 29, 30]. To further investigate this trend, we conducted supplemental viability studies (Figure S3) in which the viability of both trapped and untrapped cells was within the ideal range for DEP‐based sorting (80%–90% or higher). Although cell viability in our initial experiments was lower than desirable for DEP sorting, it is important to note that measurements were completed immediately after sorting, prior to plating. Cells subsequently proliferated and returned to a healthy state before undergoing the 14‐day differentiation process. The same was true for the supplemental viability study, although those cells were not taken through adipocyte differentiation, as the primary objective was to evaluate post‐sort viability. These findings indicate that, under optimized conditions, trapped cells can maintain high viability following sorting with the CytoChip.

Our results showed that untrapped AT‐hMSCs differentiated into adipocytes more effectively than control cells, as evidenced by phase contrast imaging and positive Oil Red O staining. This suggests that untrapped cells represent a more homogeneous population of adipocyte progenitor cells compared to the unsorted control, which likely contains a mixture of adipocyte and non‐adipocyte progenitor cells. The differentiation efficiency showed some variability across voltage and frequency conditions, but overall, the adipogenic potential of untrapped cells differed from that of the unsorted control cells. The sorting conditions implemented yielded similar percentages of trapped and untrapped cells, demonstrating the CytoChip's consistent performance in isolating subpopulations with comparable trapping efficiencies. It is worth noting that although electric field stimulation is known to promote osteogenesis and inhibit adipogenesis in hMSCs [31], the timescale and stimulation conditions during DEP sorting in this study differ significantly, making it unlikely that these effects influenced the differentiation outcomes observed here.

To our knowledge, this is the first study to utilize insulating DEP to isolate subpopulations of hMSCs at the progenitor stage prior to differentiation. Our previous findings [11] linking membrane capacitance and cytoplasm conductivity to the differentiation potential of aged hMSCs informed the design of the present study. Specifically, we selected cells at Passages P5–P7 to maximize differentiation capacity and targeted higher frequencies based on the association between cytoplasm conductivity and adipogenic gene expression. However, there remains a lack of literature focused on separating hMSCs while they are still in the progenitor state to assess their differentiation potential. Previous studies have sorted hMSCs in mixtures with other cell types or differentiated progeny, such as mixtures of immortalized hMSCs with immortalized osteoblast‐differentiated hMSCs [15], hMSCs mixed with HL‐60 cells (a human promyelocytic leukemia cell line) [16], and nucleated cells characterized as hMSCs separated from erythrocytes [32]. Although these studies demonstrate that DEP is an effective label‐free technique to isolate hMSCs or their differentiated progeny, they do not address the underlying issue of inherent heterogeneity in hMSC populations. Additionally, these studies employ traditional DEP microfluidic devices with metal electrodes integrated in the main microchannels, operating at lower voltages (∼5–15 V_pp_). In the studies by Song et al. [15] and Yoshioka et al. [16], fluorescent labels were incorporated to monitor the progression of cell sorting, effectively diminishing the label‐free advantage of DEP. Throughput, an important aspect of cell sorting, varied among these studies, with estimated rates of 300–900 cells/min [15], 35,000 cells/min [32], and 50,000 cell/min with the CytoChip. For comparison, the gold standard label‐based cell sorting technique, FACS, processes cells at rates of 300,000–900,000 cells/min [33, 34], whereas magnetic‐activated cell sorting (MACS) achieves throughputs of approximately 1,900,000 cells/min [33], highlighting the trade‐off between throughput and maintaining a label‐free approach to cell sorting.

In the context of previous studies, our research underscores the utility of DEP, and specifically insulating DEP, in generating subpopulations of hMSCs. This work contributes to the growing body of literature on label‐free technologies in stem cell research. Although our application of insulating DEP successfully sorted hMSCs at the progenitor level, challenges remain, including the need for standardized sorting protocols and comprehensive downstream analyses of sorted cells. One limitation of our study was the cell death observed within the trapped cell population, as well as the focus on adipogenic differentiation as the sole lineage investigated. Although we initially observed reduced viability among trapped cells, supplemental viability studies indicated that both trapped and untrapped cells are able to maintain high viability following insulating DEP sorting under optimized conditions. We addressed viability in the untrapped population during the main study by implementing a post‐separation process that supported proliferation and recovery, enabling successful adipocyte differentiation. However, recovery remained less effective for trapped cells in the initial experiments. Future studies could mitigate this issue by reducing the batch collection time (< 20 min) and further optimizing sorting parameters to enhance viability and maintain functionality in trapped cell populations.

Despite these limitations, our findings have several implications for harnessing DEP to produce homogeneous subpopulations of hMSCs. Stem cell therapies are increasingly being explored for treating metabolic diseases. Although hMSC transplantations have demonstrated a favorable safety profile, variability in therapeutic outcomes highlights the need for homogeneous subpopulations of cells. By generating functionally distinct progenitor populations, insulating DEP could play a crucial role in improving the consistency and efficacy of hMSC‐based therapies.

## 5 Conclusion

Insulating DEP represents a promising tool for isolating homogeneous subpopulations of hMSCs in a label‐free manner. This study demonstrates the utility of the CytoChip for separating trapped and untrapped cell populations based on differences in their DEP behavior, highlighting its potential to address the inherent heterogeneity of hMSCs. Our results show that untrapped cells exhibit enhanced adipogenic differentiation, suggesting that DEP‐based sorting can enrich progenitor populations with specific functional potentials. Future studies will build on these findings by integrating electrical property measurements with functional outcomes to strengthen the link between DEP behavior and cell phenotype. We will also incorporate molecular analyses, including immunostaining to refine subpopulation characterization and qPCR of post‐sort passaged cells to capture gene expression profiles associated with differentiation potential. These efforts, combined with expansion into additional lineages such as osteogenic, chondrogenic, and myogenic differentiation, and benchmarking against FACS as a gold standard, will further enhance the value of DEP‐based sorting for enriching functionally distinct progenitor cells.

## Conflicts of Interest

The authors declare no conflicts of interest.

## Supporting information




**Supporting File 1**: elps70001‐sup‐0001‐SuppMat.pdf.

**Supporting File 2**: elps70001‐sup‐0002‐figureS1.jpg.

**Supporting File 3**: elps70001‐sup‐0003‐figureS3.jpg.

**Supporting File 4**: elps70001‐sup‐0004‐videoS1.avi.

## Data Availability

The data that support the findings of this study are available from the corresponding author upon reasonable request.
