# Supplementary material for: Label‐Free Sorting of Human Mesenchymal Stem Cells Using Insulating Dielectrophoresis
Source: Electrophoresis. 2025 Jul 24;46(18):1438–46. doi: 10.1002/elps.70001 (PMC12532083; doi:10.1002/elps.70001)
Supplement: Supplementary file 1 — Supporting File 1: elps70001‐sup‐0001‐SuppMat.pdf. [file ELPS-46--s002.pdf]

Supplemental Information

We assessed cell morphology prior to DEP sorting by analyzing suspended hMSCs for size and circularity using hemocytometer images. The average cell radius was 10.39 ± 2.62 μm, and the average circularity was 0.48. Circularity ranges from 0 to 1, where a value of 1 represents a perfect circle.

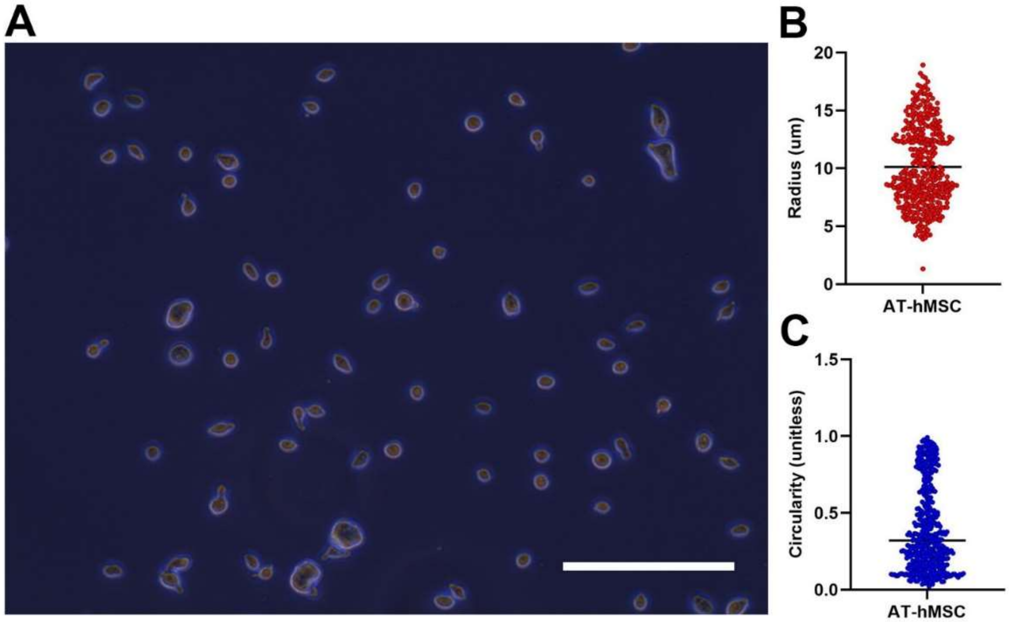

Supplemental Figure S1. (A) Representative hemocytometer image of AT-hMSCs prior to cell sorting. (B) Cell radius values. (C) Cell circularity values. Scale bar = 200 μm.

Supplemental Video S1. Demonstrates differences in velocity of a cell trapping along insulating posts (blue) and a cell remaining untrapped (green).

Viability of untrapped AT-hMSC subpopulations sorted by insulating DEP is presented in Supplemental Figure S2. While viability trends varied across different voltage and frequency conditions, untrapped cells sorted at 200 Vpp and 200 kHz exhibited the highest viability, whereas higher voltages and higher frequencies showed reduced viability without statistically significant differences.

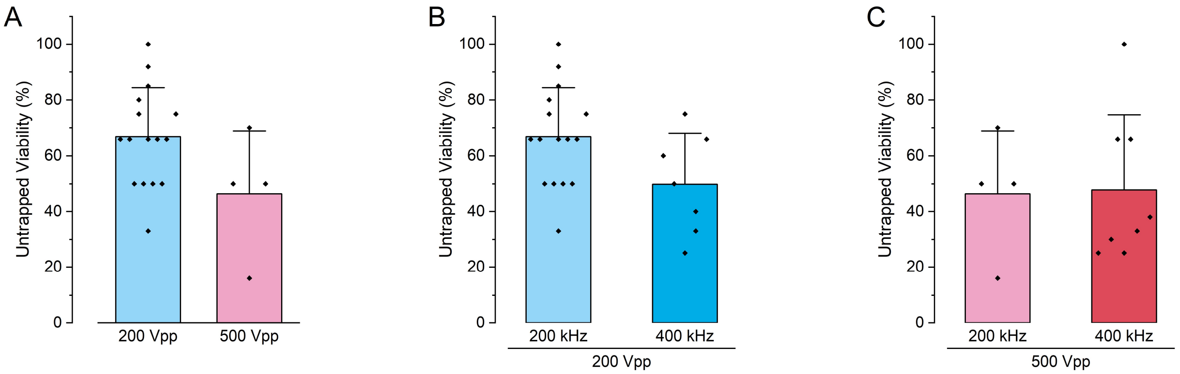

Supplemental Figure S2. Viability of untrapped AT-hMSC subpopulations sorted using insulating DEP. (A) Viability percentages for cells across a voltage sweep at 200 kHz. (B-C) Viability percentages across frequency sweeps.

Supplemental viability studies were completed at 200 kHz and 200 V_{pp} using bone marrow derived hMSCs which showed ~85% viability for both trapped and untrapped cells, compared to ~91% for unsorted controls. After post-sort passage one (PSP1), viability remained high, with ~83% for trapped cells and ~87.5% for untrapped cells.

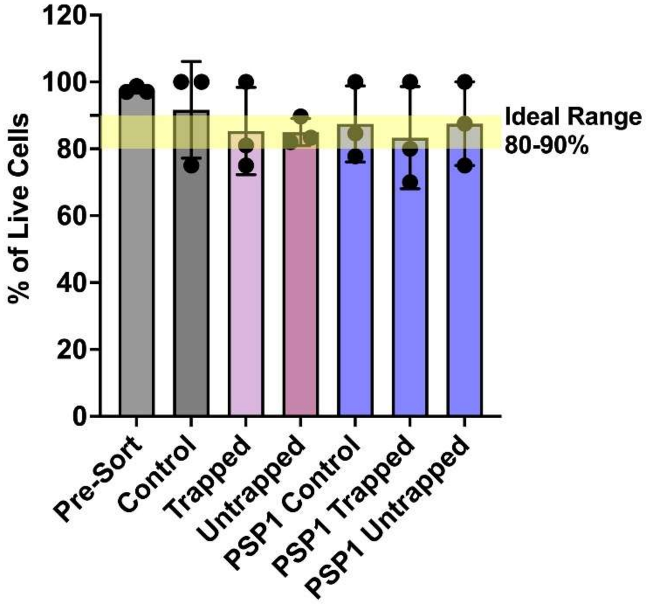

Supplemental Figure S3. Viability analysis of control, trapped, and untrapped hMSCs sorted at 200 kHz and 200 V_{pp}. "Pre-Sort" reflects cells cultured prior to sorting. "PSP1 Control," "PSP1 Trapped," and "PSP1 Untrapped" reflect cells that underwent one passage after sorting (PSP1 = Post-Sort Passage 1). The yellow shading indicates the ideal range for viability (80–90%), with higher values also considered optimal.
