# Supplementary figures and images for: Label‐Free Sorting of Human Mesenchymal Stem Cells Using Insulating Dielectrophoresis
Source: Electrophoresis. 2025 Jul 24;46(18):1438–46. doi: 10.1002/elps.70001 (PMC12532083; doi:10.1002/elps.70001)

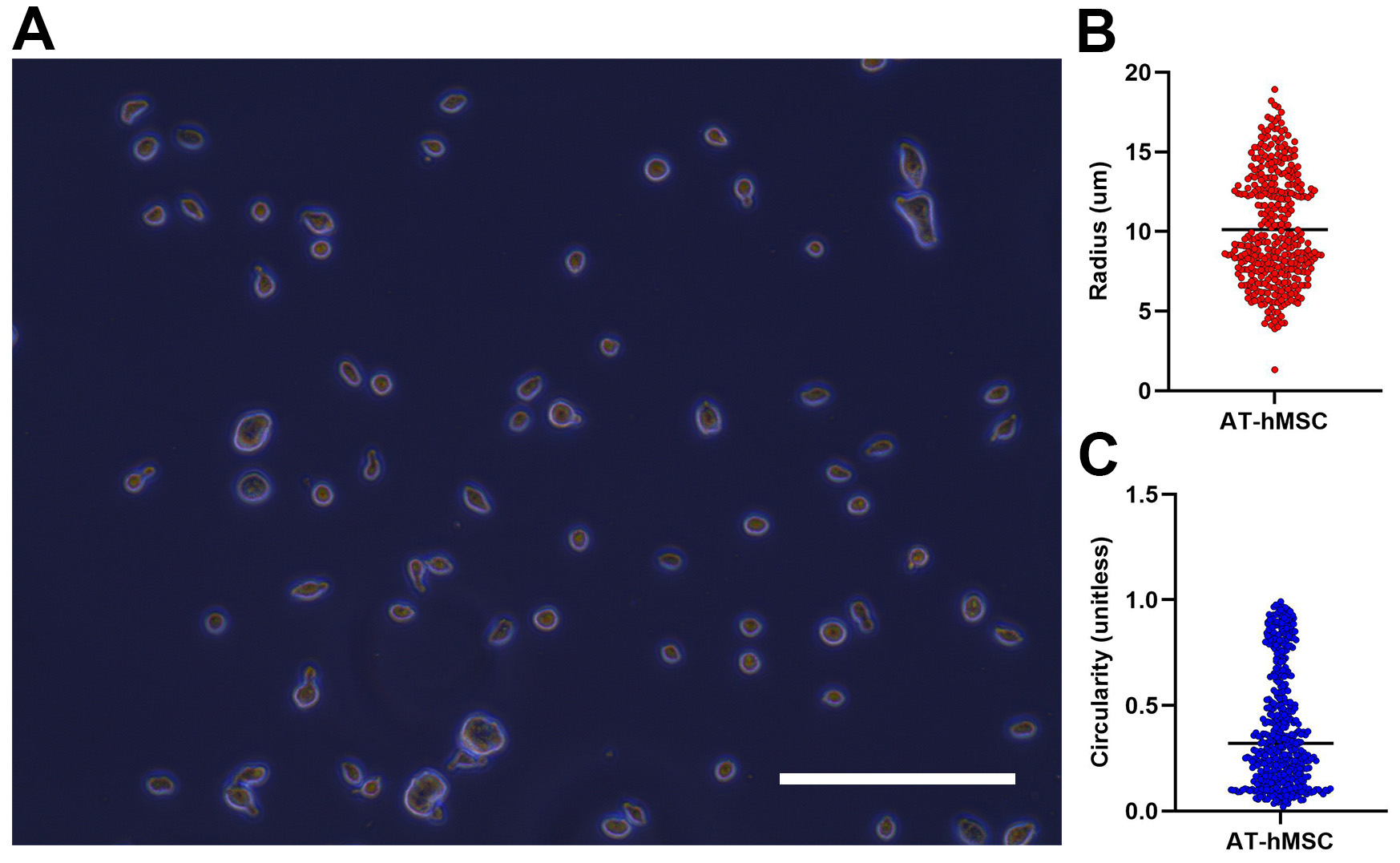

Supplement: Supplementary file 2 — Supporting File 2: elps70001‐sup‐0002‐figureS1.jpg. [file ELPS-46--s004.jpg]

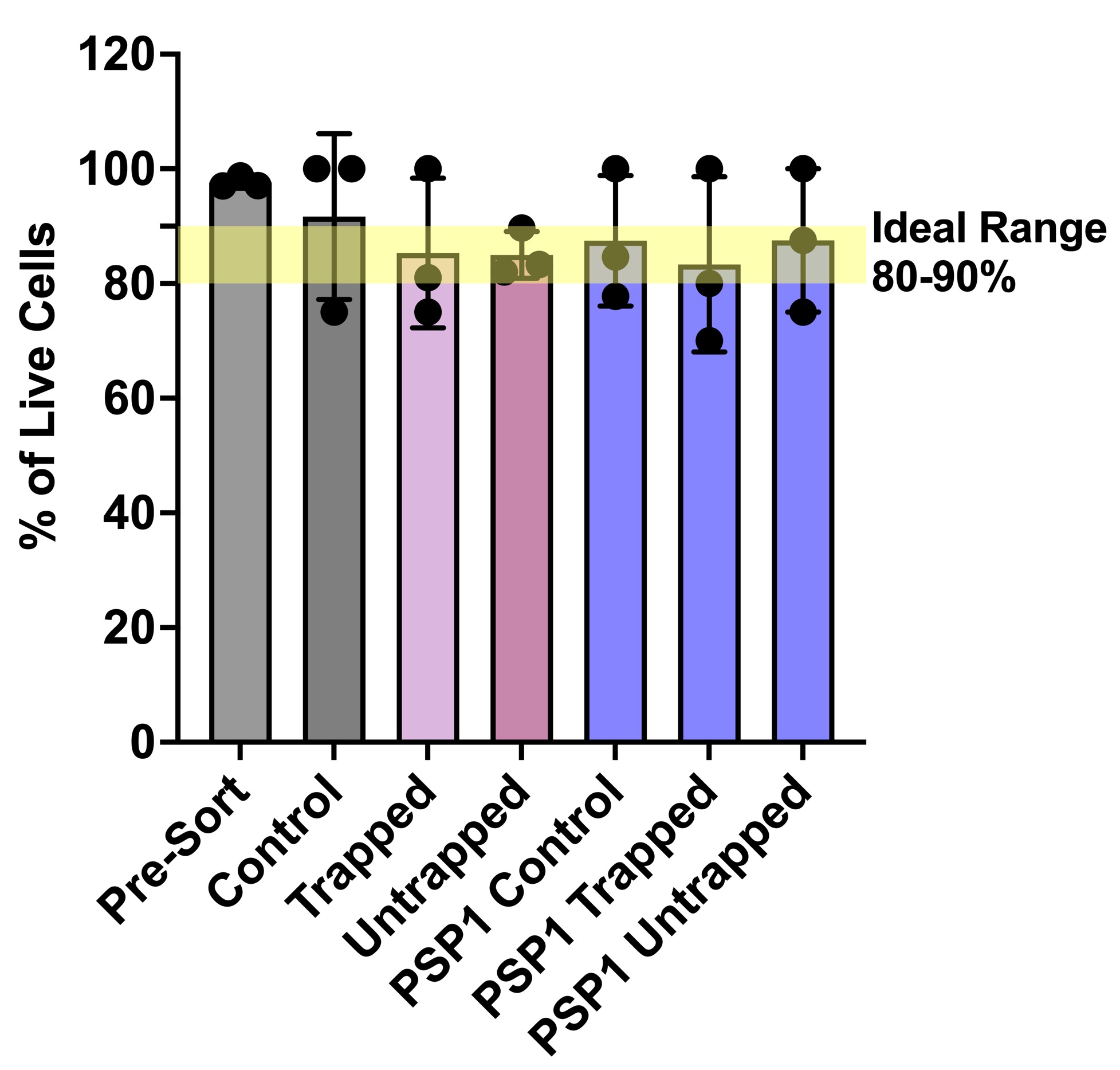

Supplement: Supplementary file 3 — Supporting File 3: elps70001‐sup‐0003‐figureS3.jpg. [file ELPS-46--s001.jpg]
